# Letter to the Editor From Janot et al: « Single-Exon Deletions of ZNRF3 Exon 2 Cause Congenital Adrenal Hypoplasia »

**DOI:** 10.1210/clinem/dgae229

**Published:** 2024-04-09

**Authors:** Clément Janot, Anne Bachelot, Delphine Mallet, Dominique Simon, Pierre Val, Florence Roucher-Boulez

**Affiliations:** Hospices Civils de Lyon, LBMMS, Service de Biochimie et Biologie moléculaire, Centre de Biologie et de Pathologie Est, Bron cedex F-69677, France; Université Claude Bernard Lyon 1, Faculté de Médecine Lyon Est, Lyon F-69008, France; Department of Endocrinology and Reproductive Medicine and Centre de Référence des Maladies Endocriniennes Rares de la Croissance et du Développement, Centre de Référence des Pathologies Gynécologiques Rares, AP-HP, IE3M, Hôpital Pitié-Salpêtrière, 75013 Paris, France; Sorbonne Université Médecine, 75006 Paris, France; Hospices Civils de Lyon, LBMMS, Service de Biochimie et Biologie moléculaire, Centre de Biologie et de Pathologie Est, Bron cedex F-69677, France; Pediatric Endocrinology department, AP-HP, Hôpital Robert Debré, 75019 Paris, France; Institute of Genetics, Reproduction and Development (iGReD), CNRS UMR6293, INSERM 15 U1103, Clermont Auvergne University, Clermont-Ferrand F-63000, France; Hospices Civils de Lyon, LBMMS, Service de Biochimie et Biologie moléculaire, Centre de Biologie et de Pathologie Est, Bron cedex F-69677, France; Université Claude Bernard Lyon 1, Faculté de Médecine Lyon Est, Lyon F-69008, France; Stem-Cell and Brain Research Institute (SBRI), Inserm U1208, 18 avenue Doyen-Lépine, 69675 Bron Cedex, France

**Keywords:** adrenal, genetics, ZNRF3

We read with great interest the article by Amano et al, which addresses single-exon 2 deletions of *ZNRF3* as a novel contributor to adrenal hypoplasia (1). The advent of next-generation sequencing technologies has facilitated genetic diagnosis for the majority of these patients. However, some individuals continue to face diagnostic hurdles, with inadequate monitoring. To underscore the significance of Amano et al's breakthrough and emphasize the importance of investigating such deletions, we present an additional case involving a de novo deletion of exon 2 in the *ZNRF3* gene.

The authors detailed the cases of 3 patients who exhibited neonatal onset of adrenal insufficiency, along with ventricular septal defect for 2 of them. Our patient is a 33-year-old French woman from a nonconsanguineous family. She experienced neonatal salt wasting at 10 days of life [ACTH 1092 pg.mL^−1^ (reference interval: 7-52), cortisol 331 pmol.L^−1^ (reference interval at 8 Am: 150-500), renin 530 pg.mL^−1^ (reference interval: 11-72)]. She was initiated on glucocorticoid and mineralocorticoid replacement, which led to resolution of her electrolyte abnormalities.

She faced diagnostic impasse since childhood, despite undergoing DNA analysis for various genes associated with adrenal insufficiency and whole-exome sequencing (WES) (2). It was only through the examination of an extended gene panel that we identified a *ZNRF3* exon 2 deletion, missed by the copy number variation calling algorithm on the WES. Our findings support the idea put forward by Amano et al that detecting single-exon deletions using methods like WES can be difficult and emphasizes the need for more sensitive techniques, like whole-genome sequencing.

Prior to the publication by Amano et al, we initially classified this deletion as of uncertain significance. However, together both reports reaffirm the crucial role of Wnt/β-catenin signaling in adrenal cortex development. The deletion of 42 amino acid derived from exon 2 hinders the binding to R-Spondin1 (RSPO1) and consequently enables ZNRF3 to evade RSPO1-dependent clearance, leading to the constitutional inactivation of the Wnt-β-catenin pathway. In light of the recent publication by Lucas et al (3), which demonstrated that inactivating LGR4, the R-Spondin receptor, in the adrenal cortex led to decreased WNT signaling and aberrant zonation, this underscores the potential for variations in other genes within the intricate network of LGR4/Wnt/β-catenin signaling to be considered.

Extra-adrenal manifestations may arise when Wnt/β-catenin signaling is impaired. Unlike the 2 Japanese patients, our patient had no cardiac issues. One may also expect gonadal defects following alteration of ZNRF3 function. Indeed, the absence of ZNRF3 results in testis determination defect because of ectopic canonical WNT signaling in XY gonads, while the loss of RSPO1 results in masculinization of XX individuals (4). However, similar to the only girl (10,8 years old) of the Japanese cohort, our patient had normal pubertal development. It started at 11 years, with the first menstrual cycle occurring at 13 years and 2 successful spontaneous pregnancies at 27 and 32. She exhibited regular menstrual cycles with normal gonadal function analysis at 26 years in early follicular phase: FSH (4.6 IU/L), LH (4.8 IU/L), estradiol (32 pg.mL^−1^), anti-Müllerian hormone (42.7 pmol/L). She has been maintained on glucocorticoid and mineralocorticoid treatment and had normal growth on −1.5 SD with no other sequelae.

Our letter highlights the continued diagnostic challenges despite advanced sequencing, emphasizes the importance of sensitive techniques, and expands the understanding of the various manifestations of *ZNRF3* gene mutations.
